# Bioenergy Crisis in Coronavirus Diseases?

**DOI:** 10.3390/brainsci10050277

**Published:** 2020-05-02

**Authors:** Anirban Dutta, Abhijit Das, Daniel Kondziella, Michal K. Stachowiak

**Affiliations:** 1 Department of Biomedical Engineering, University at Buffalo, Buffalo, NY 14260, USA; 2 Department of Neurology, The Walton Centre NHS Foundation Trust, Liverpool L9 7LJ, UK; abhijit.neuro@gmail.com; 3 Department of Neurology, Rigshospitalet, Copenhagen University Hospital, 2100 Copenhagen, Denmark; Daniel.Kondziella@regionh.dk; 4 Department of Clinical Medicine, Faculty of Health and Medical Sciences, University of Copenhagen, 2200 Copenhagen, Denmark; 5 Department of Pathology and Anatomical Sciences, University at Buffalo, Buffalo, NY 14203, USA; mks4@buffalo.edu

Coronavirus disease (COVID-19) has been declared as a pandemic by the World Health Organization (WHO). Typical symptoms reported in COVID-19 are respiratory illness. About 80% of the infections are not severe (even asymptomatic), whereas out of the hospitalized COVID-19 patients with pneumonia, about 50% developed hypoxemia by day eight while about 17–29% developed acute respiratory distress syndrome (Wuhan experience) [1]. Recently, neurological complications are increasingly reported [2], including encephalopathy in an elderly 74-year-old male [3]. The computed tomography (CT) scan of the head showed no acute abnormalities; however, the electroencephalogram (EEG) showed bilateral slowing and focal slowing in the left temporal region with sharply countered waves. This led to the possibility of subclinical seizures due to the presence of an area of encephalomalacia consistent with the prior history of embolic stroke, and the patient remained in the intensive care unit (ICU) with poor prognosis. Filatov et al. [3] found that elderly patients with such chronic conditions and with acute infections are at an increased risk of altered mental status even though COVID-19 did not cause meningitis or encephalitis in this case study. Nevertheless, Filatov et al. [3] case study highlighted the need to identify encephalopathy as a presenting sign of COVID-19, especially in cases with altered mental status including delirium, where severe cases can lead to long-term cognitive impairments.

Respiratory virus infections trigger inflammatory responses both at the site of infection (in the upper and lower respiratory tract) as well as systemically. Here, sepsis-associated encephalopathy (SAE) can be a transient and reversible brain dysfunction in patients with COVID-19, where a subgroup of critically ill patients can develop septic shock [4]. Anti-tumor necrosis factor (TNF) antibodies have been found in the blood and diseased tissues of COVID-19 patients [5]. The severity of inflammatory excess is due to the cascade of cytokine production, the cytokine storm, where TNF can act as an amplifier of inflammation [5]. Intracranial cytokine storms can result in blood-brain-barrier breakdown without direct viral invasion [6]. In fact, the endothelium is a principal organ involved in the pathogenesis of sepsis, leading to multiple organ failure [7]. The clinical spectrum of SAE can include sickness behavior, delirium, focal deficits, and coma [8]. The EEG features of SAE can include excessive theta rhythms, predominant delta rhythms, triphasic waves, and burst suppression along with seizures in up to 15% of patients. The review by Heming et al. [8] highlighted the use of various EEG monitoring tools in sepsis; however, Heming et al. [8] also found that the EEG monitoring methods remained ill-defined for sepsis. Heming et al. [8] reported that SAE is associated with neurovascular uncoupling due to microcirculatory dysfunction and low blood flow. Therefore, we postulate that the use of EEG monitoring will be more informative in conjunction with functional near-infrared spectroscopy (NIRS) such that any neurovascular uncoupling can be detected [7] during EEG events. Neurovascular coupling is important since it adapts local cerebral blood flow to the neural metabolic needs [9] that maintains the neuroenergetic status of the neurovascular tissue so any neurovascular uncoupling can lead to an energy crisis in the brain tissue [10]. Here, a majority of the energy in the brain is generated by the oxidative phosphorylation in the mitochondria where the energy currency, adenosine triphosphate (ATP), production rate plays a central role in brain bioenergetics [11]. 

Lee and Huettemann [10] presented a model in which inflammatory signaling changes the phosphorylation state of the mitochondrial proteins leading to inhibition of the oxidative phosphorylation. Since oxidative phosphorylation in the mitochondria generates a majority of the ATP so inhibition of the oxidative phosphorylation can lead to an energy currency crisis. Moreover, hypoxemia due to severe respiratory failure in respiratory virus infections can further aggravate the energy crisis. Powerful anti-inflammatory drugs can limit the inflammation but have the risk of increasing viral replication or bacterial infections [5], which can lead to meningitis/encephalitis [12]. Therefore, investigation of an adjunct therapy targeting dysfunctional mitochondrial metabolism [13] is proposed, including photobiomodulation [14], since ATP acts as a purinergic feedback signaling molecule where low ATP concentrations almost exclusively recruit microglial cells [15]. Purinergic signaling cascade is also involved with the complex vascular response at the capillaries (pericytes) [16], which can be partly responsible for the cerebrovascular complications of COVID-19 [7]. We further postulate that continuous fNIRS–EEG joint monitoring can be a useful bedside multimodal monitoring tool in neuro ICU [17] to detect transient neurovascular uncoupling. Continuous fNIRS–EEG joint monitoring will also be essential to monitor the effect of some sedative drugs that can affect neurovascular coupling and may increase the risk of delirium. However, patients in neuro ICU rarely undergo continuous brain monitoring along the lines of continuous electrocardiogram (ECG) in the cardiac ICU. Here, portable platforms with centralized multimodal data acquisition and signal processing have been found useful [18]. Moreover, some patients can be particularly susceptible to cytokine storms [19], where continuous brain monitoring can be necessary for triaging. Also, identifying genetic mechanisms underlying brain susceptibility to cytokine storms [19] will be important as predictors in addition to quantitative brain monitoring measures. Specifically, genetic insights into the mechanisms of fibroblast growth factor (FGF) signaling [20]. FGF signaling is increasingly being found essential for metabolic homeostasis in the tissues [20], where aberrant FGF receptor can enhance the Warburg Effect and mitochondrial dysfunction [21]. Recent data shows that FGF21 protects against hypoxia stress-induced injury in the cerebral microvascular endothelial cells [22]. So, FGF signaling can have a protective role not only in hypoxia-related brain disorders, e.g., encephalopathy, but also in neurodevelopmental disorders, e.g., schizophrenia [23], due to prenatal immune insult [24]. Without quantitative brain monitoring of the neuroenergetics and the functional genomics, deeper understanding of the early neurovascular signs of SAE will remain unfulfilled that is important for triaging and for tailoring the therapies. 

Mitochondrial dysfunction related to microcirculatory dysfunction [8], with an inhibition of mitochondrial respiratory chain and a decrease of oxygen utilization, remains poorly understood [25]. An increased level of proinflammatory cytokines (such as TNF, interleukins, etc.) can affect various organs by affecting their mitochondrial energy homeostasis and vascular hyperpermeability where the initial effects can be found in the skeletal muscles, heart, liver, and lungs. Here, mitochondrial respiration, which seems to evolve during sepsis [25,26], can be monitored using non-invasive broadband near-infrared spectroscopy of the cytochrome oxidase redox state [27] in various tissues including skeletal muscles. Yamane et al. [28] showed in severe influenza the relation between the host metabolic disorder-cytokine cycle and the influenza virus-cytokine-trypsin cycle in the skeletal muscles, heart, liver, and lungs (but not in the brain) which were driven by the cytokine storm. Immunomodulatory therapy has been proposed to improve the outcome in severe influenza [29]; however, its effects in the case of coronavirus disease are currently being evaluated (https://www.biocentury.com/article/304515) [30]. Nevertheless, human as well as animal studies are expensive and time-consuming so we propose a Phase-0 paradigm for drug screening and personalized medicine using microglia-containing organoid models [31,32]. This is crucial since immunomodulation can be a double-edged sword where some patients can be more susceptible than others [19]. We also propose a mini-brain computer interface (see Figure 1) [33] that combines electrophysiological recordings (using Open Ephys [34]) and Vis-near-infrared (NIR) broadband spectroscopy [35] to monitor the neuronal as well as neurometabolic coupling state in the microglia-containing cerebral-vascular organoids. Figure 1 shows the experimental setup where a 32-channel 3D microelectrode array (MEA) based electrophysiological (Ephys) recording was combined with the broadband Vis-NIR spectroscopy of the activity of the mitochondrial Electron Transport Chain (ETC) complexes. Also, computational anatomy and functional genomics were performed on the organoids [23] that are proposed to investigate genetic mechanisms underlying brain susceptibility to cytokine storms [19] and bioenergy crisis. 

In the subsequent human drug studies, broadband near-infrared spectroscopy of the brain [36] and the skeletal muscles can monitor the evolution of the systemic inflammatory response [37] to tailor the immunomodulation. An inexpensive solution using multi-wavelength continuous-wave (CW) NIRS–EEG multimodal monitoring has been developed for bedside continuous monitoring in the acute brain injury [38] to measure the neurovascular coupling (neuroenergetics) in the brain. Multiple wavelengths can be selected in the near-infrared optical windows [39] for robust CW-NIRS of the skeletal muscles and the brain where EEG in the case of the brain can provide additional metabolic disorder related features in the patients [40]. Here, the coupling relation of those EEG events, including non-convulsive status epilepticus, vis-à-vis multi-wavelength CW-NIRS-measured changes in the oxy- and deoxyhemoglobin as well cytochrome oxidase redox state can provide a marker of the severity of SAE. Therefore, we postulate that the normalization of dysfunctional EEG features as well as the neuroenergetics (from neurovascular and neurometabolic coupling) may be a prognostic marker of intact recovery without long-term cognitive impairments in the critically ill COVID-19 patients with transient and reversible brain dysfunction due to SAE. Furthermore, we highlight the need to investigate continuous bedside monitoring of bioenergetics, including mitochondrial ETC complexes, in the skeletal muscles and the brain in sepsis.

## Figures and Tables

**Figure 1:**
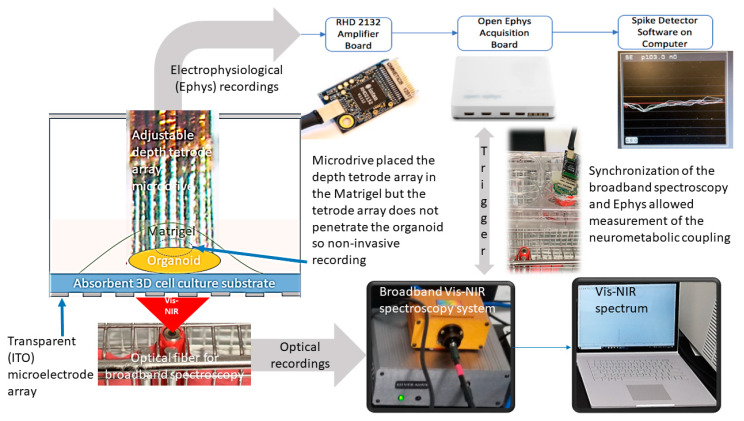
Mini-brain computer interface that combines electrophysiological recordings with the Vis-near-infrared (NIR) broadband spectroscopy to monitor the neuronal, metabolic, as well as neurometabolic coupling state in the cerebral vascular organoids (adapted from [33]).
